# Design, synthesis, characterization and computational docking studies of novel sulfonamide derivatives

**DOI:** 10.17179/excli2017-886

**Published:** 2018-02-01

**Authors:** Hira Saleem, Arooma Maryam, Saleem Ahmed Bokhari, Ayesha Ashiq, Sadaf Abdul Rauf, Rana Rehan Khalid, Fahim Ashraf Qureshi, Abdul Rauf Siddiqi

**Affiliations:** 1 Department of Biosciences, COMSATS Institute of Information Technology, Islamabad, Pakistan; 2 Department of Computer Science, Fatima Jinnah Women University, The Mall, Rawalpindi

**Keywords:** sulfonamide, derivatives, synthesis, antimicrobial, activity, structure

## Abstract

This study reports three novel sulfonamide derivatives 4-Chloro-N-[(4-methylphenyl) sulphonyl]-N-propyl benzamide (**1A**), N-(2-hydroxyphenyl)-4-methyl benzene sulfonamide (**1B**) and 4-methyl-N-(2-nitrophenyl) benzene sulfonamide (**1C**). The compounds were synthesised from starting material 4-methylbenzenesulfonyl chloride and their structure was studied through ^1^H-NMR and ^13^C-NMR spectra. Computational docking was performed to estimate their binding energy against bacterial* p*-amino benzoic acid (PABA) receptor, the dihydropteroate synthase (DHPS). The derivatives were tested *in vitro *for their antimicrobial activity against Gram+ and Gram- bacteria including *E. coli, B. subtilis, B. licheniformis *and* B. linen.* 1A was found active only against *B. linen*; 1B was effective against *E. coli, B. subtilis *and* B. linen *whereas 1C showed activity against *E. coli, B. licheniformis *and* B. linen*. 1C showed maximum activity with minimum inhibitory concentration (MIC) of 50, 100 and 150 µg/mL against *E. coli, B. licheniformis *and* B. linen* respectively. 1C exhibited maximum affinity to DHPS with binding free energy of -8.1 kcal/mol. It enriched in the top 0.5 % of a library of 7663 compounds, ranked in order of their binding affinity against DHPS. 1C was followed by 1B which showed a moderate to low level MIC of 100, 250 and 150 µg/mL against *E. coli, B. subtilis *and* B. linen* respectively, whereas 1A showed a moderate level MIC of 100 µg/mL but only against *B. linen*. These derivatives may thus serve as potential anti-bacterial alternatives against resistant pathogens.

## Introduction

Antibiotic resistance is an inevitable evolutionary phenomenon which renders antimicrobial products ineffective against infections. Antibiotic resistant microbial strains are posing substantial economic and health threats globally (Sengupta et al., 2013[12]; Lushniak, 2014[7]). Microbes have evolved various intrinsic mechanisms enabling them to develop antibiotic resistance specific to structural and functional features of antibiotics (Sengupta et al., 2013[12]). They acquire resistance against active drugs through spontaneous mutations and horizontal gene transfer. Overuse of antibiotics gradually eliminates drug-sensitive strains leaving behind drug resistant species to survive and reproduce as a consequence of natural selection (Randall et al., 2013[10]). 

Sulfonamides have long been in use as effective therapeutic agents against both Gram+ and Gram- bacterial strains associated with a range of infectious diseases (Nasr et al., 2016[9]). These broad spectrum synthetic drug molecules compete with and inhibit the binding of *p*-amino benzoic acid (PABA) in binding site of dihydropteroate synthase (DHPS) enzyme thereby interfering with the vital bacterial dihydrofolic acid synthesis pathway (Seydel, 1968[13]; Argyropoulou et al., 2009[2]). In bacteria, folic acid is an essential component required for DNA and RNA synthesis. By disrupting folic acid synthesis pathway in bacterial cell, sulfonamides compromise the microbes' ability to divide and reproduce. In addition to being antibacterial, sulfonamides and their derivatives are widely prescribed as insulin release inducers, antiviral, antifungal, anti-cancer and anti-inflammatory agents (Bano et al., 2011[3]; Zoumpoulakis et al., 2012[16]; Sławiński et al., 2013[15]). 

However, bacteria have developed resistance against popular sulfonamide based drugs such as co-trimoxazol and sulfamethoxazole etc. Owing to the rise of resistance against sulfonamides in a number of bacterial pathogens, the sulfonamides are hardly considered as first best option (Sławiński et al., 2013[15]). Growing concern associated with antibiotic resistance has spurred further research for more selective and efficacious antimicrobial solutions. To that effect, a relatively direct and cost effective strategy for drug discovery is ligand based design, which involves designing new therapeutic molecules based on structural and functional properties of already known drugs. The novel derivatives improve basic structural and functional therapeutic attributes while decoying pathogen's resistance mechanism. 

Here we report three novel sulfonamide derivatives, their mode of synthesis, physico-chemical properties, structure, binding affinities with DHPS, activity and Minimum Inhibitory Concentration (MIC) against a set of Gram- and Gram+ bacteria. These novel derivatives are listed below (Table 1(Tab. 1)). 

## Materials and Methods

### Structure of DHPS

**Figure 1:**
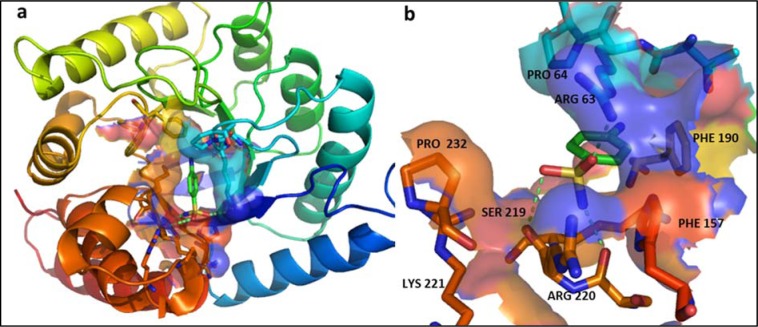
(a) Structure of *Escherichia coli* Dihydropteroate Synthase (DHPS), PDB: 1AJ0 (8). A sulfonamide molecule (C_6_ H_8_ N_2_ O_2_ S) molecule is bound inside the binding pocket. (b) Zoomed in binding pocket of DHPS shown in transparent surface, the sidechains of the residues configuring the binding pocket and imparting the polar and nonpolar interactions on sulfonamide molecule are labelled, three green dashed lines show hydrogen bonds with Arg 63, Ser219 and Arg 220.

Structure of dihydropteroate synthase (DHPS) enzyme of *Escherichia Coli, * determined through X-Ray Crystallography, solved at 2.00Å resolution (PDB: 1AJ0) already bound with a sulfonamide ligand (C_6_H_8_N_2_O_2_S) was selected as target and retrieved from RCSB's PDB database (Achari et al., 1997[1]). DHPS sulfonamide pocket is configured by H bond forming Ser219, Arg220, and Arg63 and aromatic Pro64, Phe190, Phe157 and Pro232 residues (Figure 1(Fig. 1)).

### Structure and physical properties of Sulfonamide derivatives

The structures of newly synthesized compounds were determined through NMR spectroscopy and mass spectrometry. The spectra were recorded on a Bruker DPX-400 NMR spectrometer (Billerica, USA) (400 MHz for ^1^H and 100 MHz for ^13^C-NMR), using CDCl_3_ as the solvent. The physical properties were also determined and characterized by FT-IR and Elemental analysis (CHNS). 

### Synthesis of Sulfonamide derivatives

In view of already known structure activity relationship of sulfonamide derivatives, three derivatives of sulfonamide were prepared through substitution reactions with *p-*toluene sulfonyl chloride as detailed below. 

#### Synthesis and structure of 4-Chloro-N-[(4-methylphenyl) sulfonyl]- N-propyl benzamide (1A)

**Figure 2:**
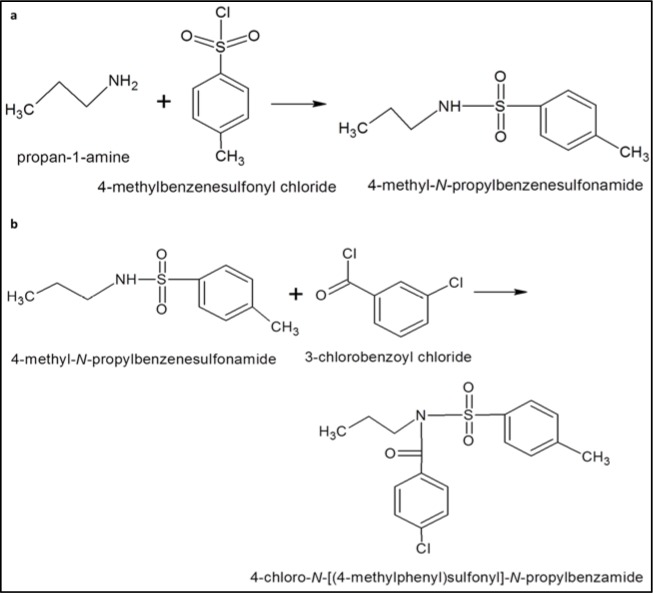
(a) Reaction of propan-1-amine with 4-methylbenzenesulfonyl chloride to produce 4-methyl-*N*-propylbenzenesulfonamide, (b) 4-methyl-N-propylbenzenesulfonamide was further reacted with 3-chlorobenzoyl chloride to obtain 4-chloro-*N*-[(4-methylphenyl) sulfonyl]-*N*-propylbenzamide.

1A was obtained by reacting propan-1-amine with 4-methylbenzenesulfonyl chloride at first followed by reaction with 3-chlorobenzoyl chloride (Figure 2(Fig. 2)). Briefly, 2.5 M solution of the propylamine (propan-1-amine) was prepared in distilled water. The solution was added to 0.44 M *p-*toluene sulfonyl chloride (4-methylbenzenesulfonyl chloride). The mixture was stirred slowly for 2-3 hours while maintaining pH during the reaction between 6-10 with 3 % Na_2_CO_3_. N-propyl benzenesulfonamide thus formed was further reacted with 3-chlorobenzoyl chloride to obtain precipitates of 4-chloro-N-[(4-methylphenyl) sulfonyl]-N-propyl benzamide. The precipitates were then filtered and washed with cold water. TLC was performed using the mixture of ethyl acetate and n-hexane (1:4) as mobile phase.

Crystalline form of compound 1A as off white powder (93 % yield) was obtained with following physical properties: molecular weight (351.85), molar refractivity (92.25±0.4 cm^3^), molar volume (275.6±3.0 cm^3^), parachor (724.3±6.0 cm^3^), index of refraction (1.58±0.02), surface tension (47.6±3.0 dyne/cm), density (1.276±0.06g/ cm^3^) and polarizability (36.57±0.5 10-24 cm^3^). NMR data of compound 1A showed ^1^H-NMR signals at δ 7.821(d, J=2.12, 1H, H-3, H-5), 7.809 (d, J=2.2, 1H, H-3′, H-5′), 7.735 (d, J=2.1, 1H, H-2, H-6), 7.351 (d, J=2.29, 1H, H-2′, H-6′), 4.207 (t, J=7, 2H, H-8) and ^13^C-NMR found signals at δ/ppm: 128.8, 128.8, 135.7, 165.7, 144.3, 56.9, 21.3, 129.7, 129.7, 45.5, 127.5, 127.5, 135.1, 128.9, 128.9, 133.7, 11.1. Elemental analysis of compound 1A found C (58.06 %), H (5.16 %), N (3.985), O (13.64 %), S (9.11 %) and Cl (10.08 %).

#### Synthesis and structure of N-(2-hydroxyphenyl)-4-methyl benzenesulfonamide (1B)

**Figure 3:**
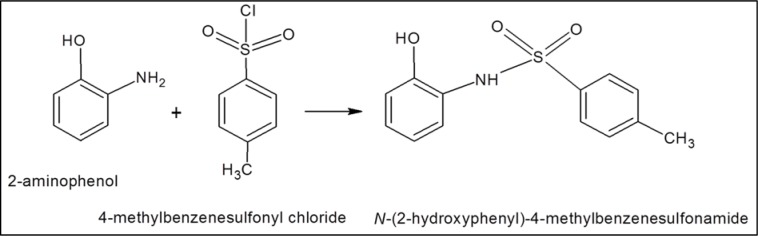
Reaction of 2-aminophenol with 4-methylbenzenesulfonyl chloride producing N-(2-hydroxyphenyl)-4-methyl benzenesulfonamide (1B).

1B was synthesized by reacting 2-aminophenol with 4-methylbenzenesulfonyl chloride, TLC was performed and the precipitates were filtered and washed with distilled cold water in a procedure similar to described earlier (Figure 3(Fig. 3)). Physical properties of white crystalline solid 1B (yield: 91 %) were: molecular weight (263.12), melting point (76 °C), molar refractivity (70.08±0.4 cm^3^), molar volume (193.2±3.0 cm^3^), parachor (538.8±6.0 cm^3^), index of refraction (1.64±0.02), surface tension (60.4±3.0 dyne/cm), density (1.36±0.06 g/cm^3^) and polarizability (27.78±0.5 10-24 cm3); ^1^H-NMR signals at δ 7.86 (d, J=8.4, 1H, H-2, H-6), 7.57 (t, J=, 1H, H-3, H-5), 7.55 (dd, J=1.8, 7.3, 1H, H-4), 7.46 (d, J=7.5, 1H, H-2′, H-6′), 7.19 (d, J=7.5, 1H, H-3′, H-5′), 2.36 (s, J); ^13^C-NMR (CDCl_3_, 100 MHz) signals at δ/ppm: 136.0, 113.7, 117.3, 127.5, 127.5, 144.3, 147.2, 129.7, 129.7, 21.3, 125.3, 131.2, 127.7. Elemental analysis of compound 1B showed C (59.30 %), H (4.98 %), N (5.32 %), O (18.23 %) and S (12.18 %).

#### Synthesis and structure of 4-methyl-N-(2-nitrophenyl) benzenesulfonamide (1C)

**Figure 4:**
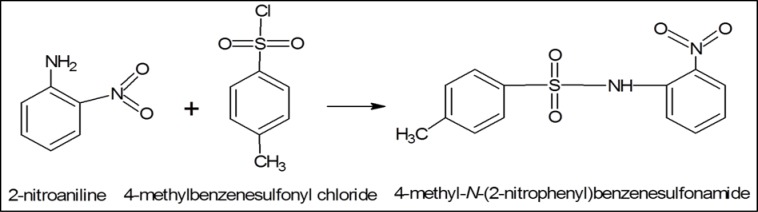
2-nitroaniline was reacting with 4-methylbenzenesulfonyl chloride to synthesize 4-methyl-N-(2- nitrophenyl) benzenesulfonamide, 1C.

In a procedure similar to that described earlier, 1C was synthesized by reacting 2-nitroaniline with 4-methylbenzenesulfonyl chloride (Figure 4(Fig. 4)), separated by TLC, crystalized, washed and dried as off-white crystalline solid (yield 93 %). Its physical properties were determined as: molecular weight (293.31), molar refractivity (74.58±0.4 cm^3^), molar volume (206.6±3.0 cm^3^), parachor (580.7±6.0 cm^3^), index of refraction (1.64±0.02), surface tension (62.3±3.0 dyne/cm), density (1.41±0.06 g/cm^3^), polarizability (29.56±0.5 10-24 cm^3^) and melting point (167 °C); ^1^H-NMR showed signals at δ 7.821 (d, J=2.12, 1H, H-3, H-5), 7.80 (d, J=2.2, 1H, H-3′, H-5′), 7.735 (d, J=2.1, 1H, H-2, H-6), 7.351 (d, J=2.29, 1H, H-2′, H-6′), 4.207 (t, J=7, 2H, H-8); ^13^C-NMR δ/ppm was: 117.3, 144.3, 127.7, 129.7, 129.7, 127.5, 127.5, 140.5, 117.3, 140.5, 127.7, 136.0, 21.3. Elemental analysis (CNHS) indicated C (53.42 %), H (4.14 %), N (9.58 %), O (21.89 %) and S (10.97 %), in the compound.

### In silico screening of sulfonamide derivatives seeded compound library

Before undertaking activity assays, we decided to screen a representative set of compounds against DHPS to see their relative binding affinity with the target.

#### Development of compound library

A diverse library of 6990 selected compounds was built from a number of open source compound libraries including compounds comparable in size, structure, physical and chemical properties to our sulfonamide derivatives. Structure files for sulfonamides 1A, 1B & 1C were written in .mol2 format and were subjected to Multiconf-Dock software (Sauton et al., 2008[11]) to generate different conformers of the synthesized sulfonamide derivatives. The conformers showed a maximum Root Mean Square Deviation (RMSD) of 2Å and free energy deviation of 10 KJ/mol from the parent structures. Thus 672 conformers of 1A, 1B & 1C were generated. These conformers along with DHPS bound sulfonamide ligand (4-aminobenzenesulfonamide, C_6_ H_8_ N_2_ O_2_ S) as in 1AJ0 PDB file were also seeded in the compound library of 6990 compounds to make up a total of 7663 compounds in .mol2 format.

#### ADMET of compound library

Absorption, Distribution, Metabolism, Excretion and Toxicity (ADMET) values of compounds were estimated based on structural characteristics or structural alerts. The compounds were rated in three categories which included: 1) those derivatives that were present in accepted range which fulfilled the physiochemical filter, 2) the compounds that lied in intermediate range i.e. showing low or very few structural alerts and 3) the compounds present in the rejected list showing high structural alerts. The last, being not suitable drug candidates, were deleted. Drug likeliness of the compounds was measured on the basis of Lipinski's rule of five (RO5). 

#### Molecular docking against DHPS

Molecular docking studies against 1AJ0 were carried out in Molecular Operating Environment (MOE) software (Chemical Computing Group ULC, 2013). Being more accurate and faster, MOE is widely used to undertake *in silico *ligand screening and to predict binding affinities between small molecules and receptor protein targets. Screened library of 7663 compounds including 672 conformers of 1A, 1B and 1C were optimized in MOE. Ligand optimization included addition of partial charges through Protonate3D tools and subsequent energy minimization of these hits by applying MMFF94X force field. Afterwards optimized ligands were added to the MOE ligand database individually for docking purpose. Similarly PDB file of *E. coli* DHPS (1AJ0) was also prepared for docking by addition of non-polar hydrogen, removal of water molecules and energy minimization. Once the receptor protein was ready, site finder tool was applied to find active site in 1AJ0 structure and an electrostatic surface map was created around it to define the docking site. Later sulfonamide derivatives containing ligand database was docked within the defined docking site of 1AJ0. This tool uses triangular matcher algorithm as a default ligand placement methods to find 1000 best conformations of subject ligands within the binding pockets of the target protein (Lengauer and Rarey, 1996[6]). These 1000 poses were rescored through London dG scoring function to select top 10 conformations per molecule. For each conformation, final binding energy, S-score and RMSD values were calculated by Generalized Born Solvation Model by keeping the active pocket residues rigid. To validate docking protocol of MOE, a test run was accomplished using the co-crystallized sulfonamide ligand bound in 1AJ0 as control.

### Procedure for determining antibacterial activity

Both Gram-, Escherica coli (E. coli), and Gram+ bacteria, *Bacillus subtilis (B. subtilis), Bacillus licheniformis (B. licheniformis) *and* Brevibacterium linens (B. linens)*, were used to determine the activity. Inocula of the microbes were prepared in sterilized LB media following standard set of protocols. Sterilized petri dishes of agar medium were prepared and sterilized. The activity was measured by well diffusion and disc diffusion method as detailed below. 

#### Well diffusion method

100 µl of inoculum of each of the microbes mentioned above was spread over the media layer of the petri dishes in triplicate with a sterile cotton swab, and were put to incubate for an hour to dry. Wells 7 mm in diameter, 20 mm apart, were casted by a sterilized pipette cut near the tip. Solution of each of the compounds 1A, 1B and 1C were prepared at a concentration of 300 µg/mL. A 300 µg/mL solution of sulfamethoxazole and nutrient broth were also prepared to serve as positive and negative control respectively. 100 µl of each of the solutions were added in wells of aforementioned culture plated petri dishes for each of the four microbe cultures in triplicate and incubated for 24 hours at 36±1 °C (Table 2(Tab. 2)).

#### Disc diffusion method

The well diffusion experiment was also repeated in disc diffusion assay. In this assay instead of wells 7 mm wide Whatman filter paper discs were used. The discs were soaked with 2 mL of each sample solution, ensuring the final dry amount of the 1A, 1B and 1C to remain same as much as poured in wells in solution form; the discs were left at room temperature until complete evaporation. Discs were then placed over the surface of agar and results were recorded.

#### Procedure for determining MIC

Minimum inhibitory concentration (MIC) was estimated through micro-dilution method. The nutrient broth solution was prepared and incubated for 48 hours. 100 mg of each of the sample compounds was solubilized. Dilutions of 0.5, 1.0, 1.5, 2.0, 2.5 and 3.0 mg/mL were prepared. 0.7 mL of media (nutrient broth) and 100 µl each of the aforementioned diluted solutions were added in different test tubes along with 200 µl each of the inoculum culture. Thus the final concentrations of 50, 100, 150, 200, 250 and 300 µg/ mL were achieved in each of the tubes. The conditions were kept same for positive and negative control. The tubes were incubated for eighteen hours. Using this methodology, lowest concentration in µg/mL units of each derivative showing inhibition in growth was estimated.

## Results and Discussion

### Structural and physicochemical characterization of Sulfonamide derivatives

The structures of newly synthesized compounds were determined through spectroscopic data of IR, ^1^H NMR, ^13^C NMR and mass spectra; the physical properties were also determined and characterized by FT-IR and elemental analysis (CHNS) as given in Materials and Methods. Drug likeliness of the derivatives was evaluated on the basis of Lipinski's RO5 in which is regarded as a rule of thumb to distinguish between drug and non-drug like compounds. It predicts the probability of success of prospective drug likeliness for the subject compound on the basis of its molecular mass (≤ 500 Dalton), hydrogen bond donors (≤ 5), hydrogen bond acceptors (≤10), lipophilicity (expressed as LogP ≤ 5) and molar refractivity (between 1-40). In order to qualify as a prospective drug, a compound should meet at least two or more aforementioned thresholds. All of our sulfonamide derivatives 1A, 1B and 1C qualified the ADMET and Lipinski's rule (RO5) criteria for their drug likeliness. 

### Activity of 1A, 1B and 1C against microbes

Antimicrobial activity of the sulfonamide derivatives 1A, 1B and 1C against a set of Gram+ and Gram- bacteria including *E. coli, B. subtilis, B. licheniformis *and *B. linens* was also determined using sulfamethoxazole as a reference. 1A was found active against *B. linens*, whereas 1B was active against *E. coli, B. subtilis* and *B. linens*. 1C was effective against *E. coli, B. licheniformis *and *B. linens *(Table 2(Tab. 2))*.*

1A showed moderate level of activity with Minimum Inhibitory Concentration (MIC) of 100 µg/mL but only against *B. linens*. 1B was active against 3 of the 4 bacterial species used in this study, namely *E. coli, B. subtilis*, and *B. linens*. However, its MIC values of 100, 250 and 150 µg/mL indicated a range of moderate to low level of activity. On the other hand, 1C showed maximum activity with MIC of 50, 100 and 150 µg/mL against *E. coli, B. licheniformis* and *B. linens,* respectively (Table 3(Tab. 3)).

### Molecular docking

The newly synthesized sulfonamide derivatives showed promising activity against Gram+ and Gram- bacteria particularly against *E. coli, *the Gram- bacterium, with the exception of 1A, which was found effective only against *B. linens*. Most effective of these derivatives was 1C with best activity (MIC 50 µg/ml) against* E. coli. *This is important because *E. coli* is known to develop resistance rapidly against antibacterial drugs. Addition of 1C in the list of drugs effective against this bacterium is quite significant. Against *B. subtilis *however, 1C showed no activity*.*

Prior to the activity assays, computational studies involving molecular docking were undertaken to investigate structural and functional basis of antibacterial activity of our newly synthesized sulfonamide derivatives by estimating *in silico *binding affinities and binding conformations. Firstly, the native sulfonamide ligand was docked against DHPS structure at MOE. The ligand occupied the DHPS binding pocket in exactly same conformation showing 0Å RMSD to the co-crystallized structure (1AJ0). Ligand conformations were same with focused docking, (when receptor binding pocket dimensions and coordinates were defined through 3D grid) and with blind docking (when binding pocket was not defined and the whole receptor structure was targeted for docking). Thus, DHPS binding pocket was found perfectly selective for sulfonamides. 

**Figure 5:**
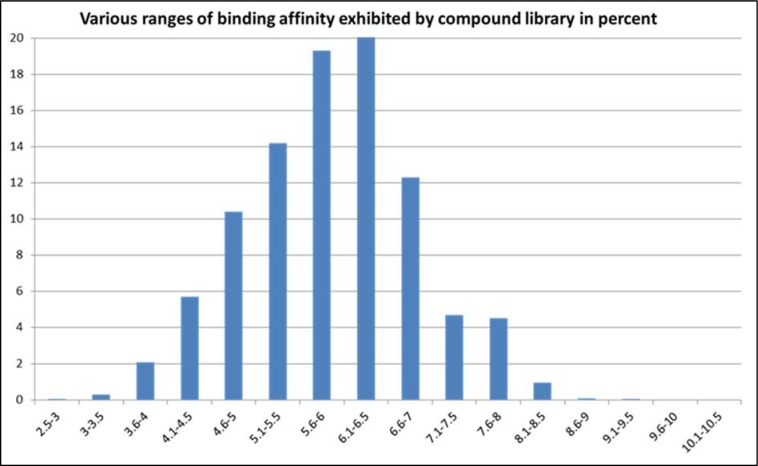
Ranges of binding energy exhibited by the compounds in the library. Each histogram represents fraction of the compound library in percent (%) exhibiting the corresponding range of binding energy at the x-axis; the values are shown in positive. 1C with a binding energy of 8.1 kcal/mol was found in the most favorable range (8.1-8.5 kcal/mol) enriching in the top 0.57 % of the library.

In the second instance, an exhaustive docking protocol was followed, which involved both blind and focused docking of our selected library of 7663 compounds seeded with 672 conformers of 1A, 1B and 1C and also the native sulfonamide ligand bound with DHPS. The docked compounds were later ranked on the basis of their binding affinity. Interestingly, derivative 1C ranked in top 40 compounds both in focused and blind docking out of a library of compounds which included popular sulfonamide drugs such as sulfacetamide, sulfadiazine, sulfisoxazole, sulfisomidine, sulfaisodimidine, sulfadoxine and sulfamethoxazole with a binding affinity of -8.1 kcal/mol (Figure 5(Fig. 5)).

**Figure 6:**
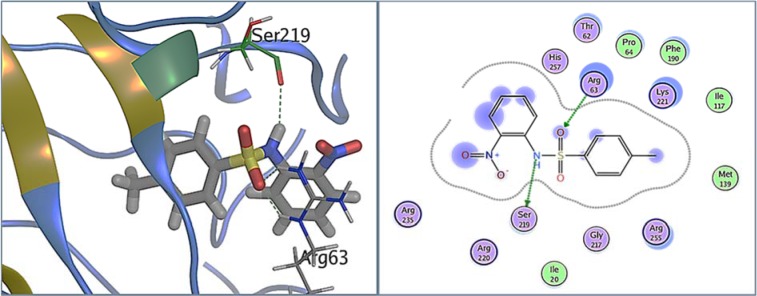
1C bound in DHPS binding pocket. A: DHPS residues exhibiting multiple interactions with 1C, inhibiting its PABA binding pocket. Green dotted lines represent hydrogen bonds between Arg63 and oxygen of sulfonyl group, and between Ser219 and -NH group of 1C. B: 2D representation of DHPS in complex with 1C. Residues are represented in three letter code with their position. Arrows in green dotted lines represent hydrogen bonds with donor at the base and acceptor at the arrow head. Polar and hydrophobic residues are shown with purple and green interiors respectively; the basic residues are shown in blue rings. Differences in solvent accessible surface area for 1C atoms and DHPS residues are plotted as blue smudge and turquoise halo, respectively.

Figure 6(Fig. 6) shows the binding pose of 1C in the pocket of DHPS. 1C exhibited strong hydrogen bonds with binding pocket residues Arg63 and Ser219 involving nucleophilic oxygen of the sulfonyl group and nitrogen of the amino group. Whereas, strong hydrophobic interaction was also exhibited by other residues of the binding pocket which include Ile20, Pro64, Ile117, Phe 190, Lys221, Ser219, Gly227 and His257. 

**Figure 7:**
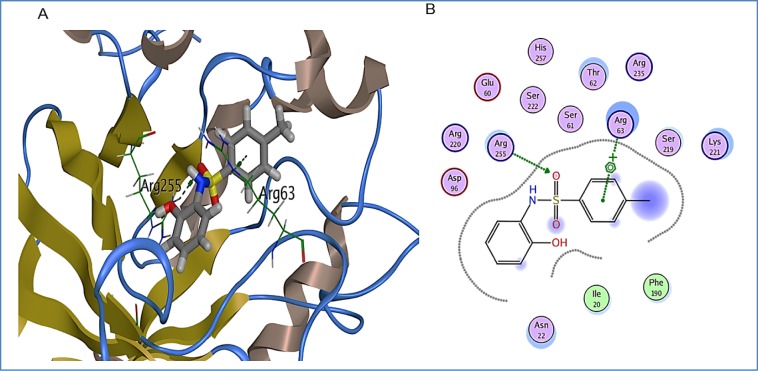
1B bound in DHPS in binding pocket. A: DHPS residues exhibiting multiple interactions with 1B. Green dotted lines represent hydrogen bond between Arg255 and oxygen of sulfonyl group of 1B and arene-cation interaction between Arg63 and aromatic ring of methyl benzenesulfonamide group. B: 2D representation of DHPS in complex with 1B. Residues are represented in three letter code with their position. Arrows in green dotted lines represent hydrogen bonds with donor at the base and acceptor at the arrow head. Polar and hydrophobic residues are shown with purple and green interiors respectively; the acidic and basic residues are differentiated by red and blue rings respectively. Differences in solvent accessible surface area for 1B atoms and DHPS residues are plotted as blue smudge and turquoise halo respectively.

1B also exhibited appreciable interaction to the binding pocket of DHPS. Strong hydrogen bond between Arg255 and the oxygen of sulfonyl group was observed. In addition to this, residue Arg63 exhibited arene-cation interaction with the methyl benzenesulfonamide group of 1B (Figure 7(Fig. 7)). Binding score of 1B with binding pocket of DHPS was observed to be -5.4 kcal/mol. Other binding pocket residues Ile20, Asn22, Glu60, Ser61, Thr62, Asp96, Phe190, Ser219, Lys221, Asp220, Ser222 and Arg235 were also found interacting with 1B.

**Figure 8:**
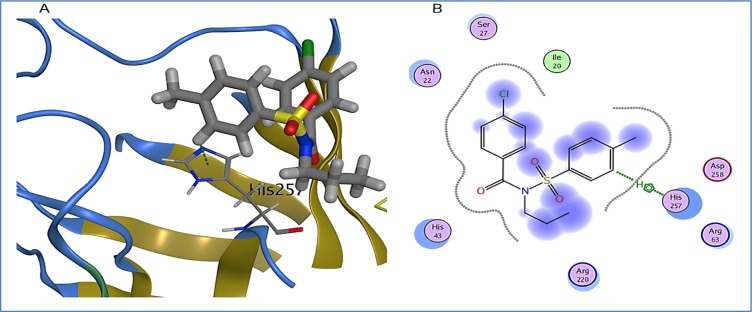
1A bound in binding pocket of DHPS. A: Green dotted lines represent arene-hydrogen bond between His257 and 1A. B: 2D representation of DHPS in complex with 1A. Residues are represented in three letter code with their position. Arrows in green dotted lines represent hydrogen bonds with donor at the base and acceptor at the arrow head. Polar and hydrophobic residues are shown with purple and green interiors respectively; the acidic and basic residues are differentiated by red and blue rings respectively. Differences in solvent accessible surface area for 1A atoms and DHPS residues are represented as blue smudge and turquoise halo respectively.

1A exhibited arene-hydrogen bond interaction with binding pocket residue, His257, while its binding energy was found to be -4.2 kcal/mol. His257 being a basic residue usually participates in hydrogen bonding by accepting protons. Other binding pocket residues exhibiting hydrophobic interactions were Ile20, Asn22, Ser27, His43, Arg63, Arg220 and Asp258 (Figure 8(Fig. 8)).

This study reports three novel sulfonamide derivatives which showed activity against both Gram+ and Gram- bacterial pathogens. 1A showed moderate level activity against *B. linens*. 1B was active against *E. coli, B. subtilis *and *B. linens* showing moderate to low level activity. 1C was the most active sulfonamide derivative with a high level activity against* E. coli *and moderate level activity against *B. licheniformis* and *B. linens* (Tables 1&2(Tab. 1)(Tab. 2)). The derivatives have demonstrated appreciable structural and functional properties to inhibit the PABA binding pocket of bacterial DHPS with optimum values of binding energy (Figures 6-8(Fig. 6)(Fig. 7)(Fig. 8)). Of a particular importance is 1C which enriched in top 0.57 % of the compound library ranked in order of the binding energy when docked against DHPS (Figure 5(Fig. 5)). Surprisingly, 1C did not show any activity against *B. subtilis.*

Owing to their prolonged usage, bacteria have developed resistance against reputed sulfonamide drugs. Thus their capacity to inhibit microbial pathways has been gradually compromised (Sköld, 2000[14]). Ligand based strategies have been widely employed to discover active sulfonamide derivatives. A number of research studies have sought to develop novel sulfonamide derivatives through structure aided optimization of already known sulfonamide scaffolds to achieve maximum biological activity and minimum microbial resistance (Ezabadi et al., 2008[4]; Kamel et al., 2010[5]; Zoumpoulakis et al., 2012[16]; Mondal et al., 2015[8]). Thus new sulfonamide derivatives may inhibit the target enzymes as well as decoy the bacterial resistance mechanisms. Designed through ligand based approach, three novel sulfonamide derivatives (1A, 1B and 1C) with substantial drug potential are presented for further *in vivo *and clinical verification. 

## Conclusion

This study describes synthesis, structure and physico-chemical properties of three novel sulfonamide derivatives with their structural and functional affinities and their MIC values against a set for Gram+ and Gram- bacteria. To the best of our knowledge, these derivatives, their synthesis and physico-chemical properties along with computational docking investigations and *in vitro *antimicrobial studies against the subject bacterial species have not been reported before. The reported derivatives exhibited significant antimicrobial properties against bacteria. 4-methyl-N-(2-nitrophenyl) benzenesulfonamide (1C) was found highly active against *E. coli *along with* B. licheniformis *and *B. linens* as indicated by its high MIC and binding energy values. This was followed by N-(2-hydroxyphenyl)-4-methyl benzenesulfonamide (1B) which showed moderate to low activity against *E. coli, B. subtilis *and *B. linens*; whereas 4-chloro-N-[(4-methylphenyl) sulfonyl]- N-propyl benzamide (1A) was found moderately active against* B. linens. *Thus 1C was found most active derivative and it may serve as an effective drug particularly against *E. coli *related pathogenesis.

## Notes

Hira Saleem, Arooma Maryam and Saleem Ahmed Bokhari equally contributed as first authors.

Fahim Ashraf Qureshi (Department of Biosciences, COMSATS Institute of Information Technology, Islamabad, Pakistan; eMail: qureshifa@comsats.edu.pk) and Abdul Rauf Siddiqi equally contributed as corresponding authors.

## Conflict of interest

The authors declare that they have no conflict of interest.

## Figures and Tables

**Table 1:**
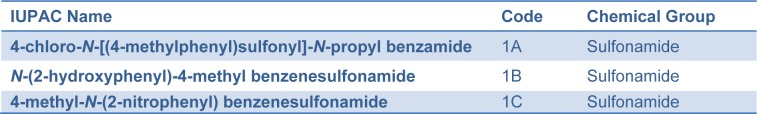
Sulfonamide derivatives synthesized in this study

**Table 2:**
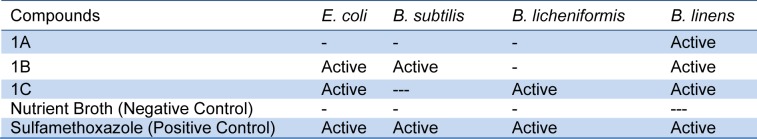
Activity of Sulfonamide derivatives 1A, 1B and 1C against bacterial pathogens *E. coli, B. subtilis, B. licheniformis *and* B. linen*

**Table 3:**
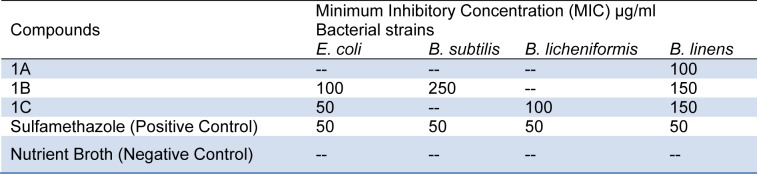
Minimum Inhibitory Concentration (MIC) of 1A, 1B, and 1C against various bacterial strains
